# Mathematical inspection of heat transfer and unsteady viscous flow in a tunnel with trapezoidal shaped slender wall

**DOI:** 10.1038/s41598-023-37816-4

**Published:** 2023-07-04

**Authors:** Muhammad Naveel Riaz Dar, Azad Hussain, Faizan Hussain, Nashmi H. Alrasheedi, Khalil Hajlaoui, Mohamed Bechir Ben Hamida

**Affiliations:** 1 Department of Mathematics, University of Gujrat, Gujrat, 50700 Pakistan; 2 Department of Mechanical Engineering, College of Engineering, Imam Mohammad Ibn Saud Islamic University (IMSIU), Riyadh, Saudi Arabia; 3 Research Laboratory of Ionized Backgrounds and Reagents Studies (EMIR), Preparatory Institute for Engineering Studies of Monastir (IPEIM), University of Monastir, Monastir City, Tunisia; 4 Higher School of Sciences and Technology of Hammam Sousse (ESSTHS), University of Sousse, Sousse, Tunisia

**Keywords:** Mathematics and computing, Applied mathematics, Computational science

## Abstract

An exploration is made to investigate numerically and theoretically the time dependent flow of blood along with heat transfer through abnormal artery having trapezoidal shaped plaque. The flow is taken to be Newtonian, laminar, unsteady and incompressible. A suitable geometrical model is constructed to simulate the trapezoidal stenosis affected artery. The governed 2-dimensional momentum and heat transfer equations are conventionalized by assuming mild trapezoidal stenosis. The renovate partial differential equations are further converted into ordinary differential equations by assist of transformations. The novelty of the work is to consider unsteady blood flow through trapezoidal shape stenosed artery. A technique of finite difference is used to discretize the updated dimensionless model numerically. Comprehensive graphical outcomes for a flow of blood are obtained. The effect of trapezoidal plaque on blood velocity, pressure and temperature are shown by surface graph inside the artery and also shown with the help of line graph.

## Introduction

The unusual narrowness in a blood artery or other tubes type structure or organs is known to be stenosis. The stenosis is sometimes called as stricture. Mostly, the stenosis cause loss of life when the narrowness crosses the significant limit of narrowing enough to disrupt the blood flow. Stenosis disrupts blood supply in human arteries, causing defiance to flow to be greater than normal. The major source of creation of this stenosis in arteries is still not known precisely but the effects can be identified freely. The significant additions of recent decade to the current topic are cited in^1–6^. A lot of investigators in this field demonstrate that the probes are mainly involved single non-symmetric and symmetric narrowness. The stenosis has different shapes in nature. Stenosis may be irregular or triangular or elliptic or trapezoidal or develop in series or composite or overlapping in nature. The power law blood flow model for overlapping stenosis is examined by Ismail et al.^7^.

In various portray, El Kot and Mekheimer^8–11^ studied particular features of flow of blood in stenotic arteries. The analysis of flow of blood for various non-Newtonian model types have been analyzed by Nadeem and Akbar^12–16^. Siddiqui and Mishra^17^ studied the blood circulation by composite stenotic artery. Mishra and Sinha^18^ explained the effect of slip velocity on flow of blood through blood vessel along with porous wall. Nadeem et al.^19^ explored the effect of nano-particles on flow of blood in stenotic artery.

The bio-heat transportation process in hemodynamics has a greater effect on the progression of atherogenic mechanisms, but they provide an interesting insight in to beneficial theoretical and empirical exploration. The recognizing the fluctuation of temperature profile as a feature of artery diameter is crucial for creating suitable bio-heat transfer models. Temperature profile is impeded in physiological scenarios whenever the radius of the blood artery is massive^20^. Based on the past literature, when the artery is extremely large, localized cooling portions are noticeable inside the heated body tissue throughout hyperthermia cure^21,22^. Human body seems to have a normal temperature of 37 °C. As a result, irreversible damage to blood proteins happens, that can lead to death with such mild fever^23^. Furthermore, hyperthermia or hypothermia is frequently used for various reasons, including cancer treatment and surgery of heart, where temperature is crucial. The magnitude of temperature is significant in hyperthermia cure because it raises the temperature magnitude of cancer affected tissues over a therapeutic value of 42 °C, whereas keeping surrounding tissue at a particular temperature magnitude. Several studies have been conducted in the past to investigate heat transportation in artery. When hyperthermia cure is considered, Zaman et al.^24^ examined the impact of heating framework on temperature variation in a single manner artery and cancer tissue. They came to conclusion that large arteries have influence on the heat transportation features of tissues undergoing hyperthermia cure. Mustafa^25,26^ examined the thermal transportation through porous channel and rotating disk. Tamunoimi and Ogulu^27^ presented the influence of heat transportation on stenotic artery under the supposition of an optically thin fluid. Turkyilmazoglu^28,29^ discussed the heat transfer for unsteady MHD fluid flow along with varying viscosity also investigated the wall heating of the square type cavity. Sarfaraz et al.^30^ investigate the influence of the different geometrical shape of stenosis on blood flow. Byoung Jin Jeon^31^ examined the trapezoidal shape stenosis effect on axisymmetric flow field around it.

So far, the past literature presented the properties and flow pattern of heat transportation on non-Newtonian and Newtonian types model. In the small artery, the blood act as a non-Newtonian at low shear rate. Moreover, it’s been revealed that the Newtonian model provides an excellent estimation while shear rates exceed 100 s^−1^, which is prevalent in large blood vessels^32,33^. Up to the understanding of authors, no detailed research is performed related to trapezoidal stenosis shape along with unsteady laminar viscous flow. So, it’s impossible to perform a comparative investigation in this direction. From this motivation, the main purpose to perform this study is to analyze the effect of trapezoidal stenosis on blood velocity, pressure and temperature. So, that we will able to examine the reasons of rupturing of artery and paralyzes of body parts.

## Problem formulation

**Figure 1:**
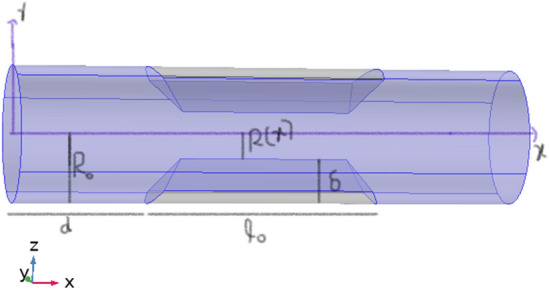
Geometrical model of stenotic artery.

The geometric model for trapezoidal shaped stenosis is developed as displayed in Fig. 1. It is clear from geometrical model that the flow is 2-dimensional in 3-D cylindrical geometry. So, the whole phenomena can be described with the assist of cylindrical coordinates $(r,\theta ,x)$. Here, r and $\theta$ are the radial and circumferential directions, and x is the axial direction. The mathematical wall geometry is given by^34^

1 $$ R\left( x \right) = \left\{ {\begin{array}{*{20}l} {\left[ {R_{0} - 2\delta \left( {x - 2d} \right)} \right]\Gamma \left( t \right), } \hfill & {d \le x \le d + l_{0} } \hfill \\ {\left[ {R_{0} - \frac{\delta }{5}\left( {\frac{{4l_{0} }}{5} - \frac{11}{5}d} \right)} \right]\Gamma \left( t \right),} \hfill & {d + l_{0} \le x \le 2l_{0} } \hfill \\ {\left[ {R_{0} - \delta \left( {1 - 2(x - 2d - \frac{{l_{0} }}{2}} \right)} \right]\Gamma \left( t \right), } \hfill & {d + l_{0} \le x \le 2l_{0} } \hfill \\ {\left[ {R_{0} } \right]\Gamma \left( t \right),} \hfill & {otherwise} \hfill \\ \end{array} } \right. $$

where $$\Gamma \left(t\right)=1-\lambda (cos\omega t-1){e}^{-\lambda \omega t}$$.

The governing compact equations for the flow of blood by trapezoidal shaped stenosis affected vessel is observed as^35^:

2 $$ \rho \frac{{\partial {\mathbf{u}}}}{\partial t} + \rho \left( {{\mathbf{w}} \cdot \nabla } \right){\mathbf{w}} = {\text{div}}\left[ { - p{\mathbf{I}}} \right] + {\text{div}}\left[ {\varvec{A}} \right] + {\varvec{F}}, $$

where $$\mathbf{A}={\upmu (\mathrm{grad}(\mathbf{w})+(\mathrm{grad}(\mathbf{w}))}^{\mathrm{T}})$$.

3 $$ \nabla \cdot \left( {\mathbf{w}} \right) = 0,\quad \left( {{\text{Incompressible}}\;{\text{flow}}} \right) $$

4 $$ \rho C_{p} \left( {\frac{{\partial {\text{T}}}}{\partial t} + {\mathbf{w}} \cdot \nabla {\text{T}}} \right) + \nabla \cdot {\varvec{q}} = Q_{p} + Q_{vd} + Q. $$

The mathematical relations used in Eq. (4) are

5 $$ {\varvec{q}} = - k\nabla T,\quad Q_{vd} = \tau \cdot \nabla w, \quad Q_{p} = \alpha_{p} T\left( {\frac{\partial p}{{\partial t}} + w\nabla p} \right), \quad \alpha_{p} = - \frac{1}{p}\frac{\partial p}{{\partial t}},\quad Q = 0 $$

where $$trace\left( {\tau \cdot \nabla {\text{w}}} \right) = \tau \cdot \nabla w$$ and $$\tau =-pI+\mu {A}_{1}$$

The above compact equations for the velocity field ${\varvec{w}}=({w}_{1}\left(r,\theta ,x,t\right),{w}_{2}\left(r,\theta ,x,t\right),{w}_{3}\left(r,\theta ,x,t\right))$ neglecting the body forces are written as:

6 $$ \frac{{\partial w_{1} }}{\partial r} + \frac{{w_{1} }}{r} + \frac{1}{r}\frac{{\partial w_{2} }}{\partial \theta } + \frac{{\partial w_{3} }}{\partial x} = 0, $$

7 $$ \rho \left( {\frac{{\partial w_{1} }}{\partial t} + w_{1} \frac{{\partial w_{1} }}{\partial r} + \frac{{w_{2} }}{r}\frac{{\partial w_{1} }}{\partial \theta } - \frac{{w_{2}^{2} }}{r} + w_{3} \frac{{\partial w_{1} }}{\partial x}} \right) = - \frac{\partial p}{{\partial r}} + \mu \left( {\frac{1}{r}\frac{{\partial \left( {rA_{rr} } \right)}}{\partial r} + \frac{1}{r}\frac{{\partial \left( {A_{r\theta } } \right)}}{\partial \theta } - \frac{{A_{\theta \theta } }}{r} + \frac{{\partial \left( {A_{rx} } \right)}}{\partial x}} \right), $$

8 $$ \rho \left( {\frac{{\partial w_{2} }}{\partial t} + w_{1} \frac{{\partial w_{2} }}{\partial r} + \frac{{w_{2} }}{r}\frac{{\partial w_{2} }}{\partial \theta } - \frac{{w_{1} w_{2} }}{r} + w_{3} \frac{{\partial w_{2} }}{\partial x}} \right) = - \frac{1}{r}\frac{\partial p}{{\partial \theta }} + \mu \left( {\frac{1}{{r^{2} }}\frac{{\partial \left( {r^{2} A_{\theta r} } \right)}}{\partial r} + \frac{1}{r}\frac{{\partial \left( {A_{\theta \theta } } \right)}}{\partial \theta } + \frac{{\partial \left( {A_{\theta x} } \right)}}{\partial x}} \right), $$

9 $$ \rho \left( {\frac{{\partial w_{3} }}{\partial t} + w_{1} \frac{{\partial w_{3} }}{\partial r} + \frac{{w_{2} }}{r}\frac{{\partial w_{3} }}{\partial \theta } + w_{3} \frac{{\partial w_{3} }}{\partial x}} \right) = - \frac{\partial p}{{\partial x}} + \mu \left( {\frac{1}{r}\frac{{\partial \left( {rA_{rx} } \right)}}{\partial r} + \frac{1}{r}\frac{{\partial \left( {A_{\theta x} } \right)}}{\partial \theta } + \frac{{\partial \left( {A_{xx} } \right)}}{\partial x}} \right). $$

By using the values of all the components, the Eqs. (7–9) are reduced as:

10 $$ \begin{aligned} & \rho \left( {\frac{{\partial w_{1} }}{\partial t} + w_{1} \frac{{\partial w_{1} }}{\partial r} + \frac{{w_{2} }}{r}\frac{{\partial w_{1} }}{\partial \theta } - \frac{{w_{2}^{2} }}{r} + w_{3} \frac{{\partial w_{1} }}{\partial x}} \right) \\ & \quad = - \frac{\partial p}{{\partial r}} + \mu \left( {\frac{2}{r}\frac{{\partial w_{1} }}{\partial r} + 2\frac{{\partial^{2} w_{1} }}{{\partial r^{2} }} + \frac{1}{r}\frac{{\partial^{2} w_{2} }}{\partial \theta \partial r}} \right. \\ & \quad \quad \left. { + \frac{1}{{r^{2} }}\frac{{\partial^{2} w_{1} }}{{\partial \theta^{2} }} - \frac{2}{{r^{2} }}\frac{{\partial w_{2} }}{\partial r} - \frac{{2w_{1} }}{{r^{2} }} + \frac{{\partial^{2} w_{3} }}{\partial x\partial r} + \frac{{\partial^{2} w_{1} }}{{\partial x^{2} }}} \right), \\ \end{aligned} $$

11 $$ \begin{aligned} & \rho \left( {\frac{{\partial w_{2} }}{\partial t} + w_{1} \frac{{\partial w_{2} }}{\partial r} + \frac{{w_{2} }}{r}\frac{{\partial w_{2} }}{\partial \theta } - \frac{{w_{1} w_{2} }}{r} + w_{3} \frac{{\partial w_{2} }}{\partial x}} \right) \\ & \quad = - \frac{1}{r}\frac{\partial p}{{\partial \theta }} + \mu \left( {\frac{2}{r}\frac{{\partial w_{2} }}{\partial r} + \frac{{\partial^{2} w_{2} }}{{\partial r^{2} }} + \frac{1}{{r^{2} }}\frac{{\partial w_{2} }}{\partial \theta }} \right. \\ & \quad \quad \left. { + \frac{1}{r}\frac{{\partial^{2} w_{1} }}{\partial r\partial \theta } + \frac{2}{{r^{2} }}\frac{{\partial^{2} w_{2} }}{{\partial \theta^{2} }} + \frac{2}{{r^{2} }}\frac{{\partial w_{1} }}{\partial \theta } + \frac{1}{r}\frac{{\partial^{2} w_{3} }}{\partial x\partial \theta } + \frac{{\partial^{2} w_{2} }}{{\partial x^{2} }}} \right), \\ \end{aligned} $$

12 $$ \rho \left( {\frac{{\partial w_{3} }}{\partial t} + w_{1} \frac{{\partial w_{3} }}{\partial r} + \frac{{w_{2} }}{r}\frac{{\partial w_{3} }}{\partial \theta } + w_{3} \frac{{\partial w_{3} }}{\partial x}} \right) = - \frac{\partial p}{{\partial x}} + \mu \left( {\begin{array}{*{20}c} {\frac{1}{r}\frac{{\partial w_{3} }}{\partial r} + \frac{{\partial^{2} w_{3} }}{{\partial r^{2} }} + \frac{1}{r}\frac{{\partial w_{1} }}{\partial x} + \frac{{\partial^{2} w_{1} }}{{\partial x^{2} }}} \\ { + \frac{1}{{r^{2} }}\frac{{\partial^{2} w_{3} }}{{\partial \theta^{2} }} + \frac{1}{r}\frac{{\partial^{2} w_{2} }}{\partial \theta \partial x} + 2\frac{{\partial^{2} w_{3} }}{{\partial x^{2} }}} \\ \end{array} } \right). $$

In present case, the velocity component $v=0,$ and flow is independent of the angle $\theta$, so, Eqs. (6) and (10–12) reduce to:

13 $$ \frac{{\partial w_{1} }}{\partial r} + \frac{{w_{1} }}{r} + \frac{{\partial w_{3} }}{\partial x} = 0, $$

14 $$ \frac{{\partial w_{1} }}{\partial t} + w_{1} \frac{{\partial w_{1} }}{\partial r} + w_{3} \frac{{\partial w_{1} }}{\partial x} = - \frac{1}{{{ }\rho }}\frac{\partial p}{{\partial r}} + \nu \left( {\frac{1}{r}\frac{{\partial w_{1} }}{\partial r} + \frac{{\partial^{2} w_{1} }}{{\partial r^{2} }} + \frac{{\partial^{2} w_{1} }}{{\partial x^{2} }} - \frac{{w_{1} }}{{r^{2} }}} \right), $$

15 $$ \frac{{\partial w_{3} }}{\partial t} + w_{1} \frac{{\partial w_{3} }}{\partial r} + \frac{{w_{2} }}{r}\frac{{\partial w_{3} }}{\partial \theta } + w_{3} \frac{{\partial w_{3} }}{\partial x} = - \frac{1}{{{ }\rho }}\frac{\partial p}{{\partial z}} + \nu \left( {\frac{1}{r}\frac{{\partial w_{3} }}{\partial r} + \frac{{\partial^{2} w_{3} }}{{\partial r^{2} }} + \frac{{\partial^{2} w_{3} }}{{\partial x^{2} }}} \right). $$

The Eqs. (4), (14) and (15) are reduced as:

16 $$ \left( {1 + 2\eta \gamma } \right)^{2} f^{\prime\prime} - \left( {1 + 2\eta \gamma } \right)ff^{\prime} + \gamma f^{2} - \left( {1 + 2\eta \gamma } \right)^{2} p_{1}^{\prime } = 0, $$

17 $$ \left( {1 + 2\eta \gamma } \right)f^{\prime\prime\prime} + \left( {f + 2\gamma } \right)f^{\prime\prime} - \frac{2\mu }{{\rho^{2} u_{0} }}p_{2} = 0, $$

18 $$ \theta^{\prime\prime}Pr = \upsilon \left( {\frac{1}{2}\theta^{\prime}\eta + 2\theta s} \right) - \frac{Pr \cdot G\upsilon }{{K\left( {T_{w} - T_{\infty } } \right)}}\left[ {\begin{array}{*{20}l} {\frac{\partial u}{{\partial r}}\left( { - p + 2\mu \frac{\partial u}{{\partial r}}} \right) + \mu \frac{\partial u}{{\partial x}}\left( {\frac{\partial u}{{\partial x}} + \frac{\partial w}{{\partial r}}} \right) + \frac{u}{r}\left( { - p + 2\mu \frac{u}{r}} \right)} \hfill \\ { + \mu \frac{\partial w}{{\partial r}}\left( {\frac{\partial u}{{\partial x}} + \frac{\partial w}{{\partial r}}} \right) + \frac{\partial w}{{\partial x}}\left( { - p + 2\mu \frac{\partial w}{{\partial x}}} \right)} \hfill \\ \end{array} } \right]. $$

The transformation used to transform the heat transfer equation is^34^:

19 $$ \left. {\begin{array}{*{20}l} {w_{1} = - \frac{1}{r}\frac{\partial \psi }{{\partial x}}, \quad w_{3} = \frac{1}{r}\frac{\partial \psi }{{\partial x}},\quad p = \frac{{u_{0} }}{l}\mu \left( {p_{1} + x^{2} p_{2} } \right)} \hfill \\ {\psi = a\sqrt {u_{w} \upsilon x} f\left( \eta \right), \quad \eta = \frac{{r^{2} - a^{2} }}{2a}\sqrt {\frac{{u_{w} }}{\nu l} } , \quad w_{1} = - \frac{1}{r}\frac{\partial \psi }{{\partial x}} = - \sqrt {\frac{{u_{0} }}{\nu l} } \frac{af\left( \eta \right)}{r} ,\quad w_{3} = \frac{1}{r}\frac{\partial \psi }{{\partial x}} = \frac{{u_{0} \nu }}{l} f\left( \eta \right)} \hfill \\ \end{array} } \right\} $$

$$T - T_{\infty } = \left( {T_{w} - T_{\infty } } \right)\theta$$, where $$\left( {T_{w} - T_{\infty } } \right) = \left( {T_{0} - T_{\infty } } \right)\frac{{x\left( {1 - st^{*} } \right)^{ - 2} }}{L}$$ and $$t^{*} = \left( {{\Omega }sin\alpha^{*} } \right)t,$$

20 $$ Pr = \frac{k}{{\rho c_{p} }},\quad G = \left( {\Omega sin\alpha^{*} } \right)^{ - 1} \left( {1 - st\left( {\Omega sin\alpha^{*} } \right)} \right)\quad {\text{and}}\quad \eta = \frac{{x\left( {\Omega sin\alpha^{*} } \right)^{0.5} }}{{\upsilon^{0.5} \left( {1 - st\Omega sin\alpha^{*} } \right)^{0.5} }}. $$

In above equations, ∇T is change in temperature, T is absolute temperature, ${T}_{w}$ shows the temperature of wall, ${T}_{\infty }$ denote free stream temperature, ${c}_{p}$ shows the specific heat at pressure, η is the dimensionless parameter, $Pr$ is Prandtl number, $\theta$ is non-dimensional temperature, k gives thermal conductivity, ${\alpha }^{*}$ is the inclined angle which is zero here, because cylinder is horizontal, Q is the source of heat without viscous dissipation, τ denotes the stress tensor and ${Q}_{vd}$ is the source of heat with viscous dissipation, ${\alpha }_{p}$ is the rate of change of pressure corresponding to absolute temperature. The similarity in Eq. (20) has been used to transform the energy equation at fixed pressure to get the required equation of heat transfer for incompressible fluid (18).

### Initial and boundary conditions

Initially, there is no flow so the velocity components ${w}_{1}={w}_{2}={w}_{3}=0$. The boundary wall has no slip, i.e., the velocity is null within wall.

Inlet of stenotic artery: $u=-{U}_{0}{\varvec{n}}$, where $U_{0} = 0.12\;{\text{m}}\;{\text{s}}^{ - 1}$, which is velocity of normal inflow.

$$ \begin{aligned} & q = h \cdot \left( {T_{ext} - T} \right),{\text{where}}\;q\;{\text{is}}\;{\text{a}}\;{\text{convective}}\;{\text{heat}}\;{\text{flux}}\;{\text{and}}\;{\text{h}}\;{\text{is}}\;{\text{heating}}\;{\text{transfer}} \\ & {\text{coefficient}}{.} \\ \end{aligned} $$

Outlet of stenotic artery: $\left[-p{\varvec{I}}+{\varvec{K}}\right]{\varvec{n}}=-\widehat{{p}_{0}}{\varvec{n}}$, where $\widehat{{p}_{0}}\le {p}_{0}$**.**

Where $p_{0} = 16000\;{\text{pa}}$ is taken for suppress backflow. Because average normal pressure of blood flow in human artery is 16 kilo-pascal.

## Numerical method

**Figure 2:**
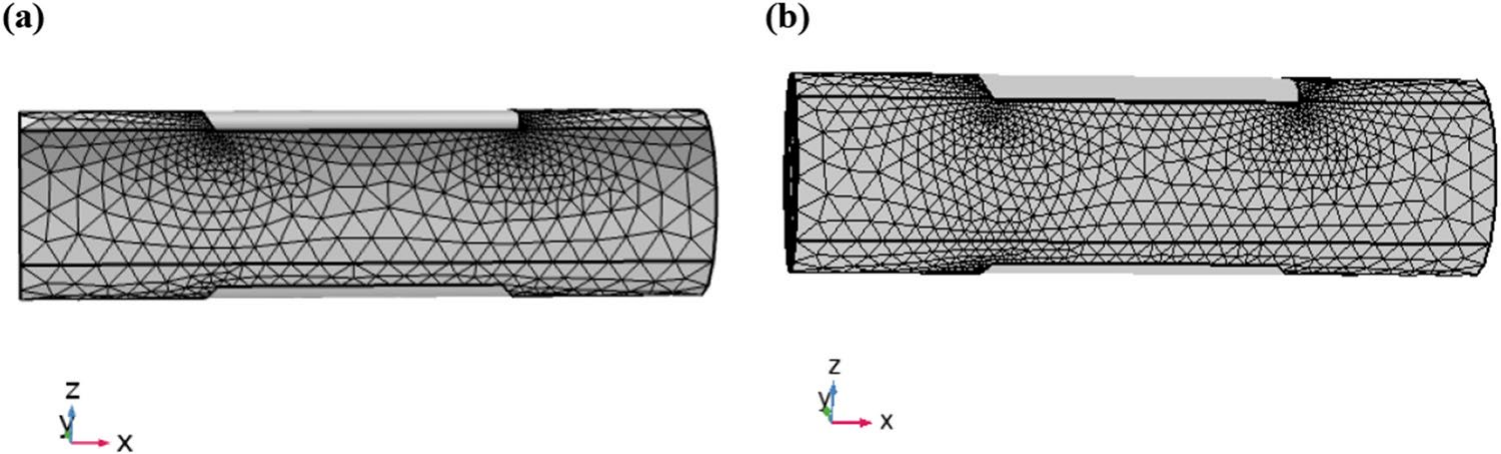
Grid illustration of geometry (coarse and normal).

The lack of any other type of solution, we chose numerical method established on finite element discretization. This element discretization consists of shape functions that possesses 9 degrees of freedom for velocity and 3 local degrees of freedom for pressure approximation^36,37^. The direct type solver is iterated after linearizing the existing nonlinear algebraic system. Grid refinement is achieved. In order to take good results, we take two grids as in Fig. 2a and 2b^38^ (Coarse and Normal) in such a way that the Degrees of freedom are compatible. Tables 1 and 2 are the mesh statistics for coarse and normal refinement which is obtain from the element discretization.

**Table 1:** Meshing statistics for coarse refinement.

Levels	DEs	BEs
No. of elements	32,740	3588
Mini. quality	0.07137	0.4252
Ave. quality	0.6486	0.8556
Ele. Volume (area)	0.1664 m^3^	2.218 m^2^

**Table 2:** Meshing statistics for normal refinement.

Levels	DEs	BEs
No. of elements	72,436	5884
Mini. quality	0.1463	0.5365
Ave. quality	0.56583	0.8683
Ele. Volume (area)	0.1669 m^3^	2.21 m^2^

## Discussion and outcomes

**Figure 3:**
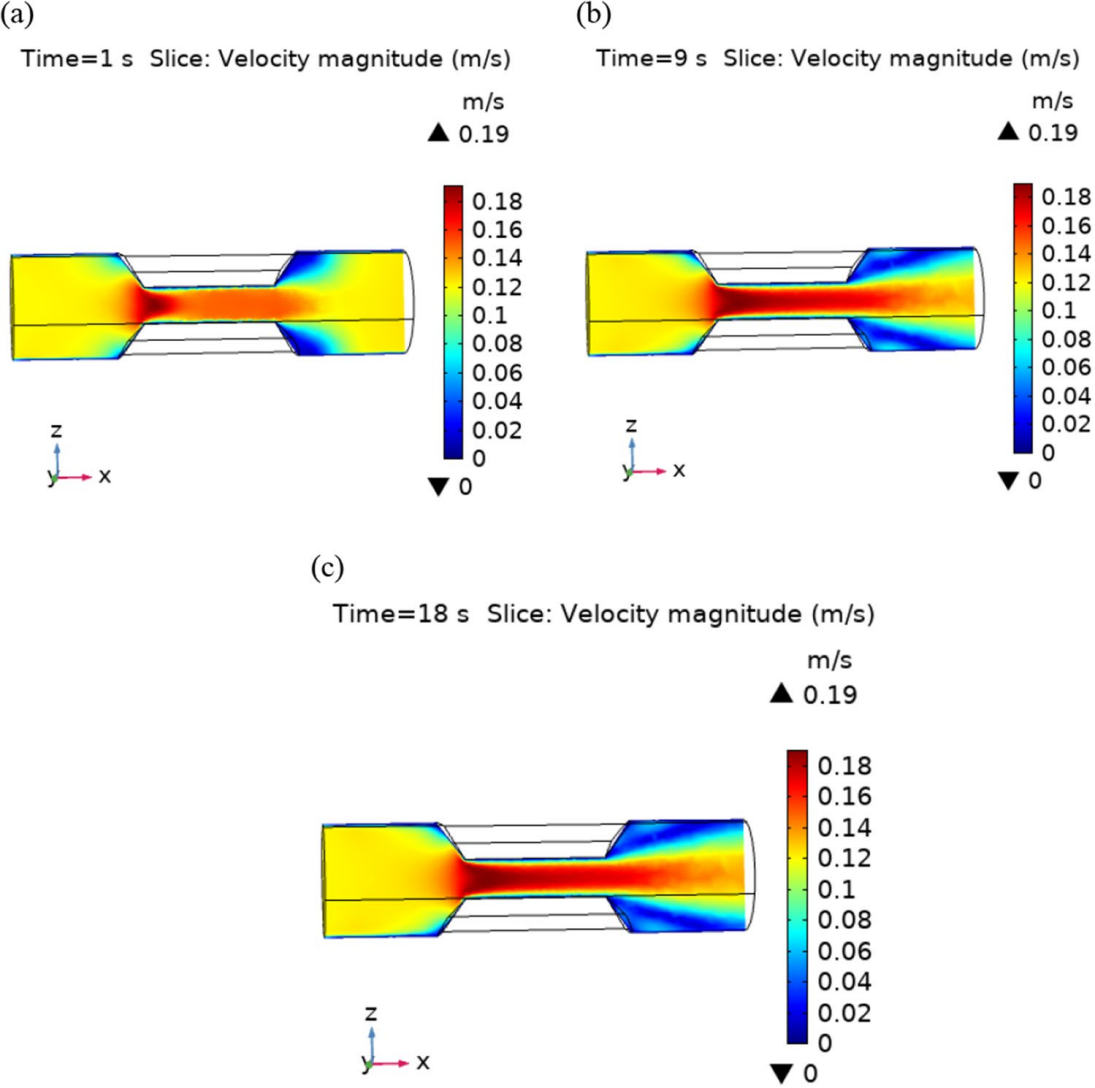
Velocity profiles for blood flow inside stenosed artery for Normal refinement at $t = 1,9 \;{\text{and }}\;18$ s.

**Figure 4:**
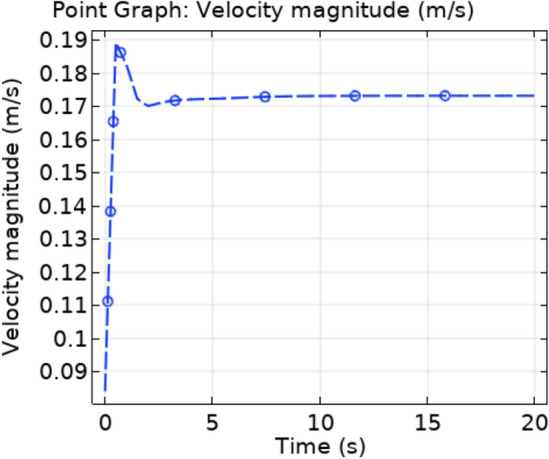
Velocity line graph with time.

**Figure 5:**
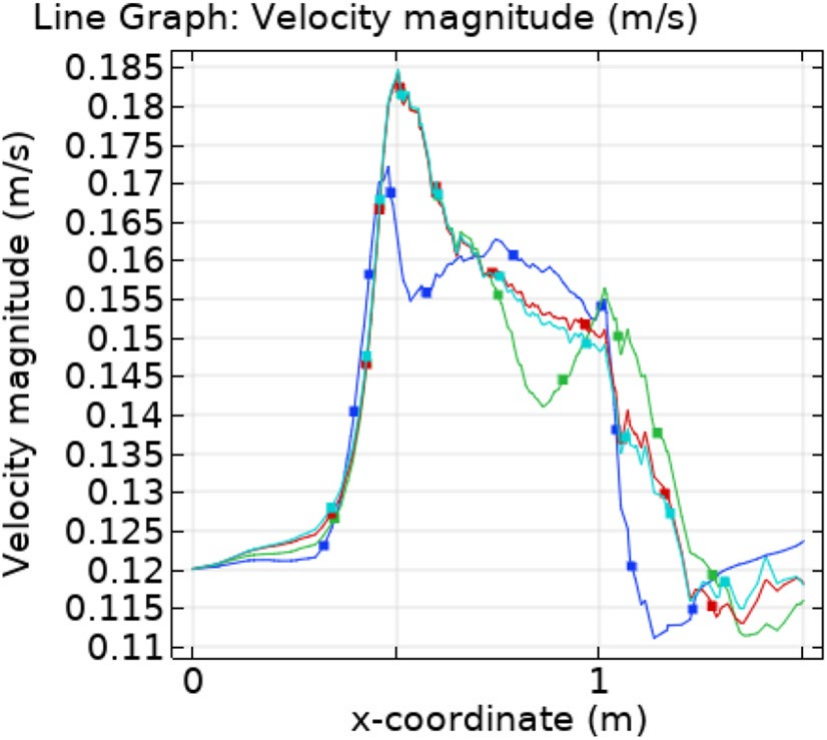
Velocity profile for different value of t along x-axis.

The trapezoidal shaped plaque is considered inside the artery. The no slip condition is applied on the boundary of plaque. The blood velocity at inlet is 0.12 ms^−1^ and convective heat flux $q = h \cdot \left( {T_{ext} - T} \right)$, where h is the heat transfer coefficient and ${T}_{ext}$ is the external temperature. A comprehensive computation is performed for different parameters with different values that explain the flow behavior in order to gain a clear understanding of the behavior of velocity, temperature, and pressure fields for Newtonian blood supply, and indeed the findings are presented graphically. The Fig. 3a–c are the velocity profiles of blood flow inside the trapezoidal stenosis affected artery. Initially, the velocity is maximum at the point where the trapezoidal stenosis is just started. After some time, this behavior is shifted throughout the stenotic region. The velocity is increasing parabolically throughout the stenosed region. Before and after the stenotic region the velocity of blood is normal. Figure 4 shows the point graph, as a point is fixed in the stenotic region and graph shows how the velocity is changing with time at that point. Figure 5 represents the line graphs for different value of “t” along the axis of flow. It can be seen that the velocity is increasing from 0.12 to 0.185 ms^−1^ at the point of stenosis and after that it is continuously decreasing.

**Figure 6:**
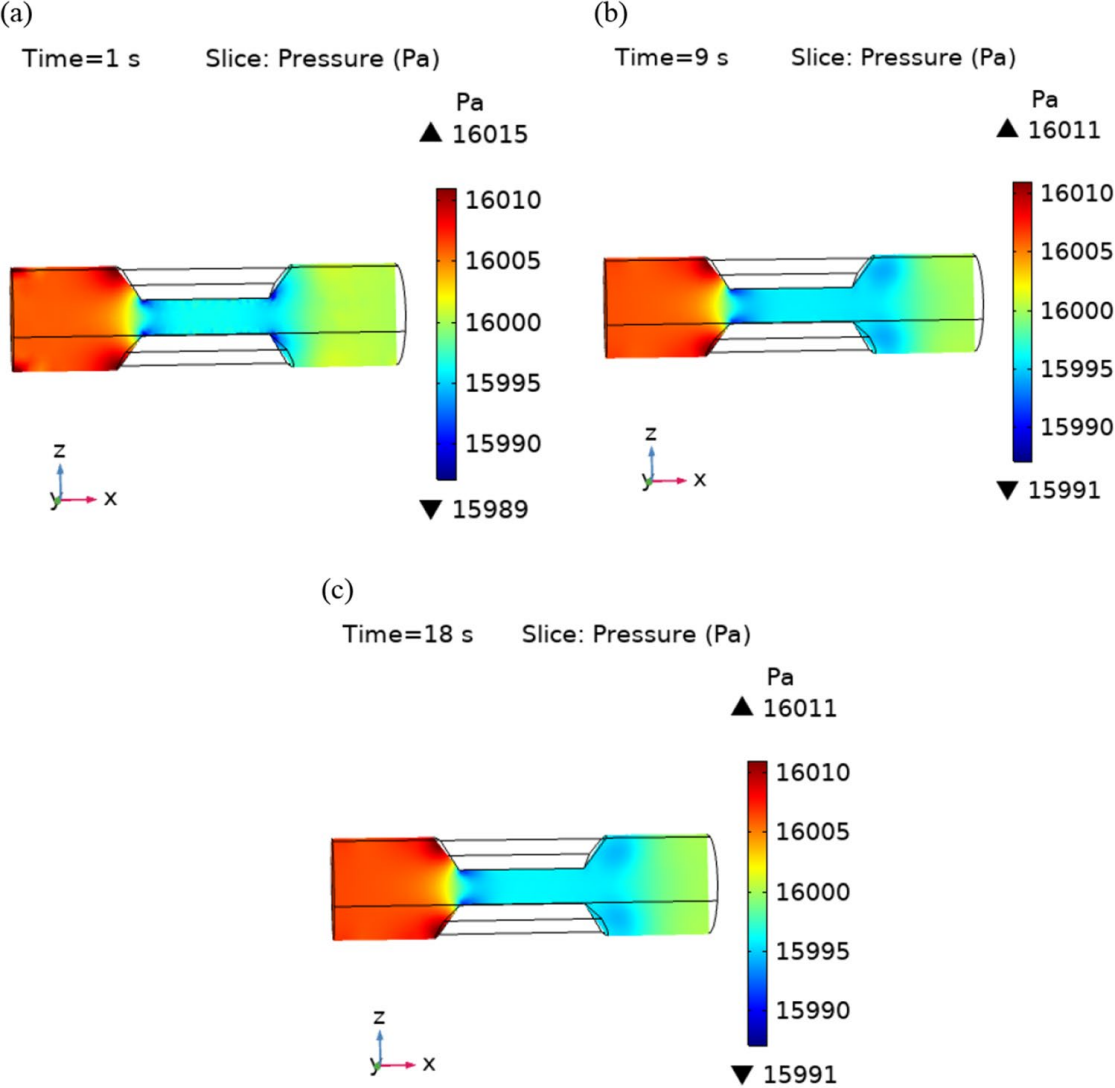
Velocity profiles for blood flow inside stenosed artery for Normal refinement at $t = 1,9 \;{\text{and }}\;18$ s.

**Figure 7:**
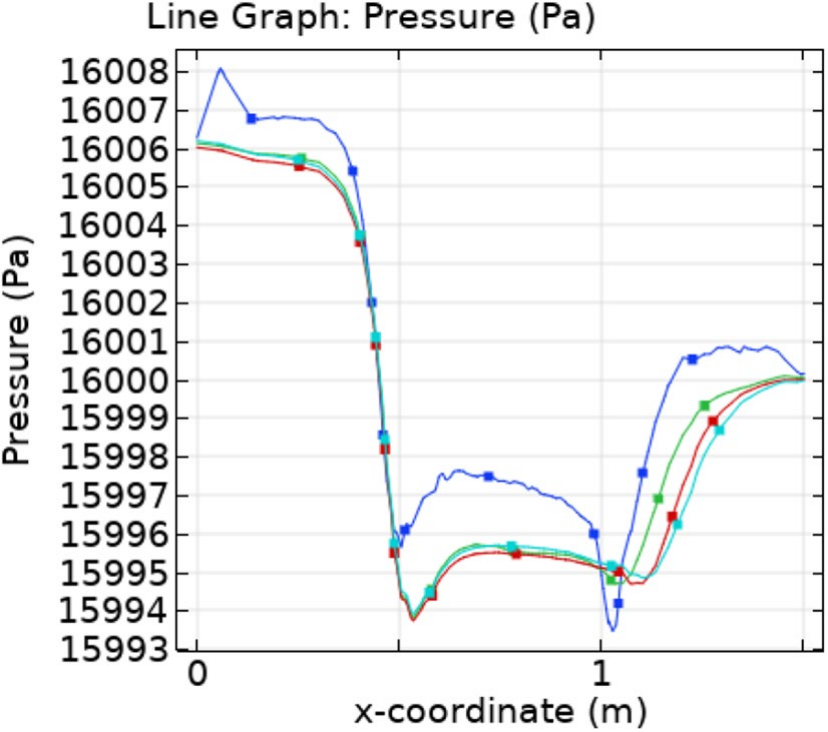
Pressure profile for different value of t along x-axis.

The Fig. 6a–c are the pressure surfaces inside the trapezoidal stenotic artery. The graph show that the maximum value of pressure is decreasing with time. The pressure is maximum before the plaque due to the narrowness of the blood path. Figure 7 displays the line graph for pressure profiles at different times along the flow axis. Initially, the pressure slightly increases and after that it decreases continuously in the stenosed region. After the stenosed region it is again increasing.

**Figure 8:**
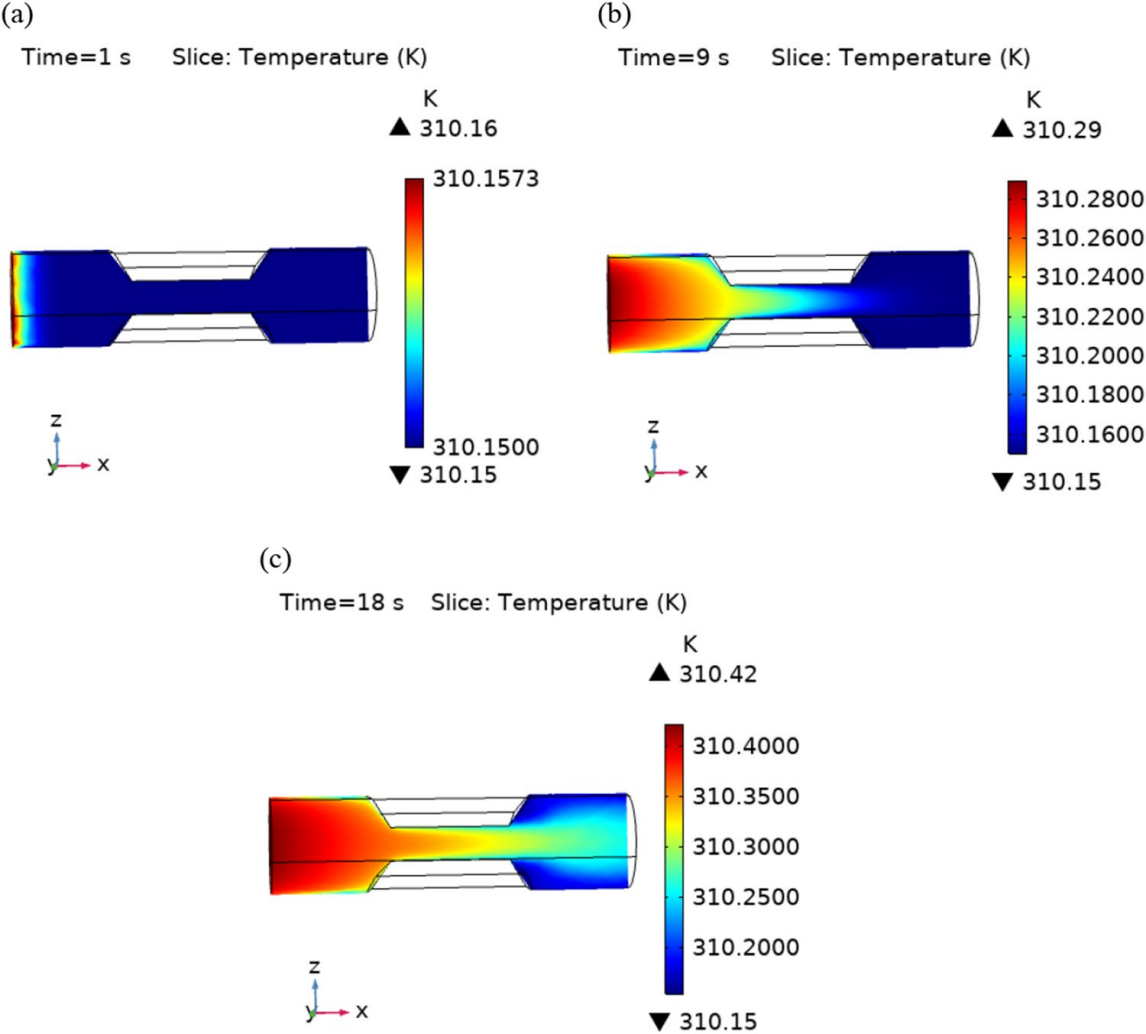
Velocity profiles for blood flow inside stenosed artery for Normal refinement at $t = 1,9 \;{\text{and }}\;18$ s.

**Figure 9:**
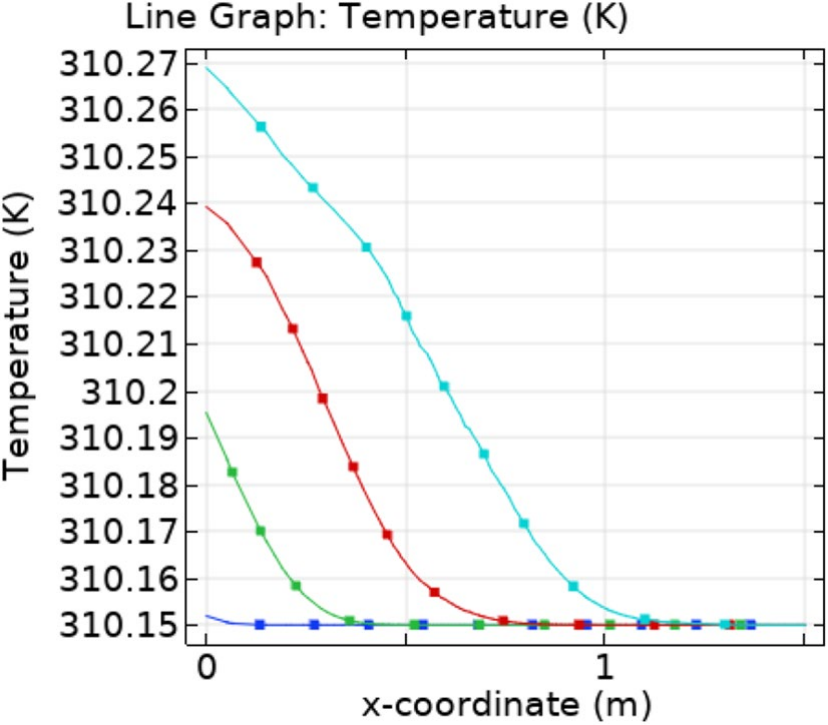
Temperature profile for different values of t along x-axis.

The Fig. 8a–c are the graphs of temperature profiles that shows the flow of heat flux throughout the stenotic region. The value of maximum temperature is continuously increasing with time. Figure 9 represents the temperature line graphs at different times along axis of flow. It can be seen that the temperature decreasing along the axis of flow for different value of “t”.

**Figure 10:**
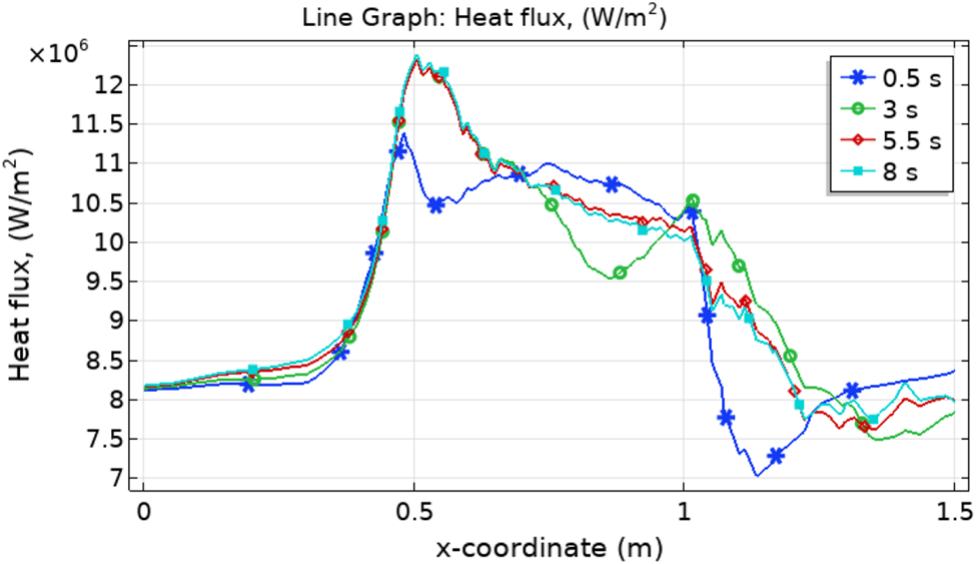
Heat flux for different values of t along x-axis.

**Figure 11:**
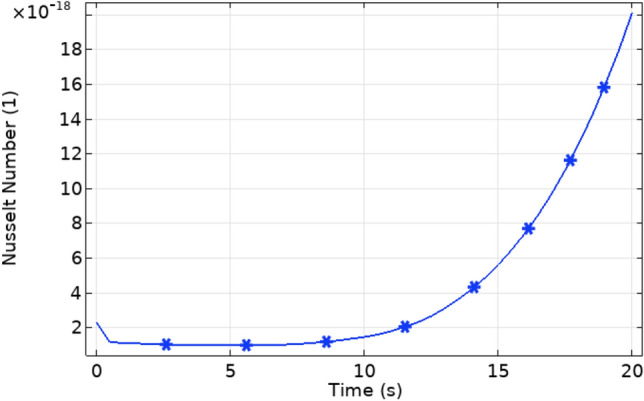
Nusselt number variation with time.

Figure 10 represents the heat flux along the axial direction. It is clear that the heat flux is maximum at the start of trapezoidal stenosis. Figure 11 shows the variation in Nusselt number. The Nusselt number increases after the stenosis region continuously which shows that heat transfer is maximum after the stenosed region.

## Validation of the model

The code in present case for the incompressible and unsteady Navier–Stokes equations established on the FEM^39^ has been validated by examining the standard experiment assisted by Ojha et al.^40^. Jung et al.^41^ investigated a finite volume method (FVM) which is based on the SIMPLER type algorithm, and a specific finite difference scheme (FDS) was utilized by Banerjee et al.^42^. The present mesh/time-step dependent study was conducted on the basis of these methods. Since there is no study have been performed for fixed shape of trapezoidal stenosis so, there is no result validation.

## Conclusion

The computational study is performed to evaluate the impact of trapezoidal shaped stenosis on the flow of blood and analyzed the affected parameters. From the graphical findings it is noted that the trapezoidal stenosis disorders the flow of blood in human artery. The simulation performed can be used in medical for better understanding of plaque. A number of the research results include as follows:

- The study of laminar blood flow revealed that the blood velocity varies in the stenotic region because of the trapezoidal arterial plaque. The maximum value is 0.185 m s^−1^.
- Initially, the velocity is maximum at the region where the stenosis is just started. But with time these maximum velocities spread in the whole stenosed region.
- The pressure profiles exemplify the clear behavior close to the trapezoidal stenotic region. The pressure is maximum just before the stenosis which cause the rupture of artery and it is minimum in the stenosed region and in the region just after the stenosis which may paralyzed that part of body.
- The temperature profile changes by changing slightly the value of time.
- We further can investigate the material characteristics, like as the skin friction coefficient, as well as analyze problems using magnetohydrodynamic and radiation impacts to determine the causes of stenosis, which might also assist in the diagnosis of vascular stenosis.

## Data Availability

The datasets used during the current study available from the corresponding author on reasonable request.

## List of symbols

${\varvec{\mu}}$: Fluid dynamic viscosity $\left( {{\text{kg}}\;{\text{m}}^{ - 1} \;{\text{s}}^{ - 1} } \right)$
x: Axial direction (m)
$${\varvec{t}}$$: Time
${{\varvec{V}}}_{0}$: Normal inflow velocity $\left( {{\text{m}}\;{\text{s}}^{ - 1} } \right)$
$${{\varvec{w}}}_{1}$$: Radial velocity $$\left( {{\text{m}}\;{\text{s}}^{ - 1} } \right)$$
$${{\varvec{w}}}_{2}$$: Tangential velocity $$\left( {{\text{m}}\;{\text{s}}^{ - 1} } \right)$$
p: Pressure of blood (kg m^−1^ s^−2^)
r: Radial direction
$$\rho $$: Fluid density
**v**: Fluid velocity field $\left( {{\text{m}}\;{\text{s}}^{ - 1} } \right)$
$\theta$: Tangential direction (rad)
$${{\varvec{w}}}_{3}$$: Axial velocity $$\left( {{\text{m}}\;{\text{s}}^{ - 1} } \right)$$
